# 545 “After The Fire: Legacy of A College Dormitory Fire Twenty Years Later"

**DOI:** 10.1093/jbcr/irac012.173

**Published:** 2022-03-23

**Authors:** Kathe M Conlon, Michael A Marano, Robin A Lee

**Affiliations:** The Burn Center as Saint Barnabas, West orange, New Jersey; The Burn Center at Saint Barnabas, Livingston, New Jersey; The Burn Center at Saint Barnabas, Livingston, New Jersey

## Abstract

**Introduction:**

In January 2000 fire erupted at a local college. Panicked students, many who failed to attend school-sponsored fire drills or simply ignored alarms, found themselves scrambling to escape. Seven students were admitted to a burn center (BC) for critical burns, with 54 initially staged in the Medical Center Emergency Department. While this dormitory fire took place more than twenty years ago, its legacy is still being felt today.

**Methods:**

History shows that, with any tragedy, there are lessons to be learned to lessen the impact on property destruction, injury or death. Looking at the legacy of this fire, four key areas of improvement emerged: disaster preparedness, media relations, legislation and fire prevention.

**Results:**

Changes to disaster preparedness include an expanded, more comprehensive response plan, revised triage and transfer protocols, BC staffing protocols, creation of a regional group of BCs, which eventually morphed into a Disaster Consortium, and a medical command center for regional disaster response. A series of articles resulted in a Pulitzer Prize-finalist book, documenting the journey of two of the survivors, with award-winning photographs displayed at a national museum. Redesigned fire safety programs emphasizing escape plans and the dangers of false alarms now targets high school and college students, an often overlooked group, and clinical education programs have expanded to include nursing’s role in disasters, reinforced with a functional exercise. Two survivors are motivational speakers, continuing to share their personal story on campuses across the United States. New legislation mandated sprinkler installation in dormitories nationwide, and a non-profit foundation was formed to improve burn care. The anniversary of this fire is commemorated annually on campus to serve as a powerful reminder for new generations of students.

**Conclusions:**

Despite this dormitory fire being ranked as the deadliest in state history, all these years later the legacy of this landmark event remains one of triumph and resilience, as its impact is still evident today. These lessons serve as the foundation for improved disaster response, locally, regionally and nationally.

